# Strategic Cell-Cycle Regulatory Features That Provide Mammalian Cells with Tunable G1 Length and Reversible G1 Arrest

**DOI:** 10.1371/journal.pone.0035291

**Published:** 2012-04-23

**Authors:** Benjamin Pfeuty

**Affiliations:** Laboratoire de Physique des Lasers, Atomes, et Molécules, CNRS, UMR8523, Université Lille 1 Sciences et Technologies, Villeneuve d'Ascq, France; Virginia Tech, United States of America

## Abstract

Transitions between consecutive phases of the eukaryotic cell cycle are driven by the catalytic activity of selected sets of cyclin-dependent kinases (Cdks). Yet, their occurrence and precise timing is tightly scheduled by a variety of means including Cdk association with inhibitory/adaptor proteins (CKIs). Here we focus on the regulation of G1-phase duration by the end of which cells of multicelled organisms must decide whether to enter S phase or halt, and eventually then, differentiate, senesce or die to obey the homeostatic rules of their host. In mammalian cells, entry in and progression through G1 phase involve sequential phosphorylation and inactivation of the retinoblastoma Rb proteins, first, by cyclin D-Cdk4,6 with the help of CKIs of the Cip/Kip family and, next, by the cyclin E-Cdk2 complexes that are negatively regulated by Cip/Kip proteins. Using a dynamical modeling approach, we show that the very way how the Rb and Cip/Kip regulatory modules interact differentially with cyclin D-Cdk4,6 and cyclin E-Cdk2 provides to mammalian cells a powerful means to achieve an exquisitely-sensitive control of G1-phase duration and fully reversible G1 arrests. Consistently, corruption of either one of these two modules precludes G1 phase elongation and is able to convert G1 arrests from reversible to irreversible. This study unveils fundamental design principles of mammalian G1-phase regulation that are likely to confer to mammalian cells the ability to faithfully control the occurrence and timing of their division process in various conditions.

## Introduction

**Figure 1:**
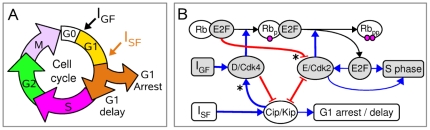
CKI-dependent regulation of mammalian G1-phase progression. (A) Cells leaving G0 following growth-factor stimulation (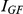) and exposed to stress/differentiation signals (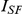) during G1 phase may be driven towards either one of two alternative fates: either G1-phase elongation or G1-arrest, which can be reversible or irreversible. (B) G1-phase progression in the presence of both 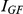 and 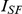 signals relies on a tight competition between two major players: 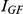-induced cell-cycle activators and 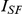-induced cell-cycle inhibitors. Main positive regulators (Grey ellipses) are the G1-specific, D- and E-type cyclins together with their favorite CDK partners and one subfamily of transcriptional factors termed activator E2Fs, which ultimately trigger S-phase entry. Negative regulators (White ellipses) include the unphosphorylated and hypophosphorylated Rb proteins and the members of the Cip/Kip family of CKIs (p21Cip1, p27Kip1, p57Kip2). Note that cyclin D-Cdks and cyclin E-Cdks are differentially regulated by unphosphorylated Rb and Cip/Kip proteins (see asterisks).

Living systems are born to reproduce and the most important challenge individual cells are faced with in their life is to decide whether and when it is time to divide. This decision is usually made during G1 phase (the lag phase that separates mitosis from the initiation of DNA replication) of the cell-division cycle, shortly before S-phase entry, at a specific ‘Start’ point in budding yeast [1], called restriction (R) point in animal cells [2], beyond which cells are irrevocably committed to divide independently of exogenous cues. While S-phase entry relies on the abrupt accumulation of active cyclin E-Cdk2 complexes in the nucleus, eukaryotic cells have evolved two major mechanisms to delay and prevent G1/S transit [3]: (i) downregulation of cyclin synthesis; (ii) inhibition of the cyclin E-Cdk2 activity by association with Cdk inhibitory proteins (CKIs). The first mechanism, which primarily operates in response to growth-factor withdrawal, induces a reversible quiescent (G0)-like phenotype. The second one, which is activated in response to a wide diversity of endogenous and exogenous signals, delays progression through G1 phase and may lead to reversible or irreversible G1 arrest (Fig. 1A). CKIs that share the same ability to enforce G1-phase delay or arrest in response to stress and differentiation signals are present in most, if not all, eukaryotic cells even though their primary structure may widely diverge amongst species [4]–[8].

In multicellular organisms like mammals, cell division actively takes place during development and tissue regeneration. This is no longer true, however, in most fully-developed organs in which local and systemic controls restrain cell division in order to maintain tissue homeostasis and prevent the emergence of cancer [9], [10]. There is clear evidence that interaction between the two G1-specific activatory modules, cyclin D-Cdk4,6 and cyclin E-Cdk2, and CKIs plays a paramount role in mammalian G1-phase control. It is still obscure, however, what particular features of this interaction might enable mammalian cells to precisely control in a contextual manner the length of their G1 phase [11], [12] and, ultimately, make the right decision regarding the occurrence of one amongst its many possible outcomes, i.e. cell division, differentiation, senescence or death [13]. The mammalian G1 regulatory network presents two striking designs that, conceivably, could participate in these events. First, cyclin D-Cdk4,6 and cyclin E-Cdk2 are activated sequentially during G1-phase progression owing to the fact that cyclin E transcription is repressed by unphosphorylated Rb proteins via the mobilization of chromatin-modifying factors and is relieved following partial Rb phosphorylation by cyclin D-Cdk4,6 [14], [15]. Second, CKIs of the Cip/Kip family that accumulate in response to stress or differentiation signals exert an opposite effect on cyclin D-Cdk4,6 and cyclin E-Cdk2 as they facilitate the activity of the former complexes while they inhibit the activity of the latter ones [16]–[18]. In this paper, we thus addressed the following questions: How does the singular organization of the mammalian G1 regulatory network determine the rate of G1-phase progression and shape the properties of G1 arrest? More generally, are there specific decision-making strategies encoded at the level of this sophisticated molecular network organization?

To answer these issues, we used a modeling approach that has proved useful to unveil design principles of molecular networks, especially those involved in cell-cycle regulation [19]. Yet, because our interest was more specifically focusing on the G1-phase period, a model of the whole cell cycle was not necessarily of use [20], [21]. That is why, we built and analysed a molecular network model limited to the cell-cycle period going from G0 exit to S-phase entry [22]–[26]. A major improvement of our model on previous ones in the field lies in the fact that it incorporates some detailed features of the interaction between the G1-specific cyclin-Cdks and the Rb/E2F and Cip/Kip regulatory modules. The model does not only reproduce the typical, previously-described properties of G1/S transition, including discreetness and irreversibility, but it also reveals how stockpiling of the Cip/Kip proteins in response to stress signals impinges on G1-phase progression such as to endow mammalian cells with the ability to easily adjust the length of their G1 phase and sustain a reversible G1 arrest. Consistently, we found that reducing the selectivity of inhibitory controls over cyclin D-Cdk4,6 and cyclin E-Cdk2 precludes long-lasting G1 phases and converts the reversible G1 arrests into irreversible ones. We further stress that these poles apart types of cell-cycle arrest correspond to two qualitatively distinct decision-making scenarios in terms of dynamical system theory [27].

## Results

### A core model of the mammalian G1-phase regulatory network

#### The eukaryotic cell-cycle machinery

Eukaryotic cell cycle progression basically relies on a tight competition between two major players: cell-cycle activators and inhibitors. The model interaction graph shown in Figure 1B incorporates the main positive and negative regulators of G1 phase. Among cell-cycle activators are the cyclins D and E together with their Cdk4,6 and Cdk2 partners, respectively, and one subfamily of transcriptional factors termed activator E2Fs, which stimulate the transcription of genes involved in both cell division, notably cyclin E, and cell death [28]. The cell-cycle inhibitors included in our model are the members of the retinoblastoma (Rb) protein family that bind to and inhibit the activator E2Fs and the members of the Cip/Kip family of CKIs (p21Cip1, p27Kip1, p57Kip2).

#### Activatory and inhibitory G1-phase regulatory signals

Synthesis and accumulation of cell-cycle activators and inhibitors require the activation of two distinct types of pathways : (i) on the one hand, the continuous provisioning of growth factors (that is pooled into the control parameter 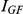, where the GF index stands for growth factors) facilitates cyclin D synthesis and accumulation and the formation, activation and nuclear accumulation of cyclin D-Cdk4,6 complexes; (ii) on the other hand, genotoxic and cytotoxic stresses (e.g. DNA damage, depletion of nucleotide triphosphates, hypoxia, nutrient deprivation, cell-cell contact, oncogenic signals, cell deformation, …) as well as differentiation signals (that are pooled into the control parameter 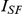, where the SF index stands for stress factors) facilitate the accumulation of CKIs. The present model does not take into account the influence of cell size and cell growth although cyclin-Cdks have been acknowledged both to regulate and be regulated by cell growth [29]–[31]. This is because we are interested here in understanding the behaviour of cells from multicelled species in which G1-phase progression is not limited by cell growth but rather by exogenous and endogenous stress signals [9].

#### Selective interactions between Rb proteins and G1-specific cyclin-Cdks complexes

Exit from G0 in mammalian cells is contingent upon growth factor-induced accumulation of cyclin D-Cdk4,6 whose first mission is to initiate Rb phosphorylation and, thereby, relieve the transcriptional repression of cyclin E genes by the Rb-E2F complexes [14]. Then, besides initiating the assembly and activation of replication complexes [32], the emerging cyclin E-Cdk2 complexes play a critical role, consisting in phosphorylating the Rb proteins whereby they free the activator E2Fs that activate a cohort of cell cycle-regulating genes and promote G1/S transit. Rb proteins, thus, can exist under three different phosphorylated forms, each of which exerts unique activities [15], [33], [34] : (1) unphosphorylated, they act as transcriptional repressors by inhibiting the activity of all three RNA polymerases [35], but also by selectively inhibiting the transcription of a number of genes, including cyclin E but not cyclin D, via the mobilization of chromatin-modifying factors [36]; (2) when partially (hypo)phosphorylated by cyclin D-Cdks, they lose their ability to directly repress transcription, including that of cyclin E; (3) when hyperphosphorylated by cyclin E-Cdk2, they dissociate from the E2F factors, enabling them to stimulate the transcription of genes involved in both cell division and cell death, of which cyclin E.

#### Selective interactions between Cip/Kip proteins and G1-specific cyclin-Cdks

Cell-cycle arrest in G1 phase is mediated in great part by the p21/p27 members of the Cip/Kip family of CKIs, which bind to and inhibit the activity of all cyclin-Cdk1,2 complexes. The Cip/Kip proteins, in turn, are quickly downregulated upon phosphorylation by the cyclin-Cdk1,2 complexes, which indicates a strong mutually-antagonistic interaction between these two components. The interaction between Cip/Kip proteins and cyclin D-Cdk is more versatile and subject to controversy. On the one hand, Cip/Kip proteins bind to the cyclin D-Cdk complexes that they assemble and target to the nucleus without inhibiting their kinase activity [16], [17]. On the other hand, it has been reported recently that context-dependent tyrosine-dephosphorylation of p27Kip1 can turn their activatory role into an inhibitory one [37], [38]. In our standard model of G1-phase, Cip/Kip will be considered as an activator of cyclin D-Cdk although the alternative scenario will be also investigated upon modification of the model.

### Cip/Kip-mediated inhibition of cyclin E-Cdk2 delays S-phase entry and induces G1 arrest

**Figure 2:**
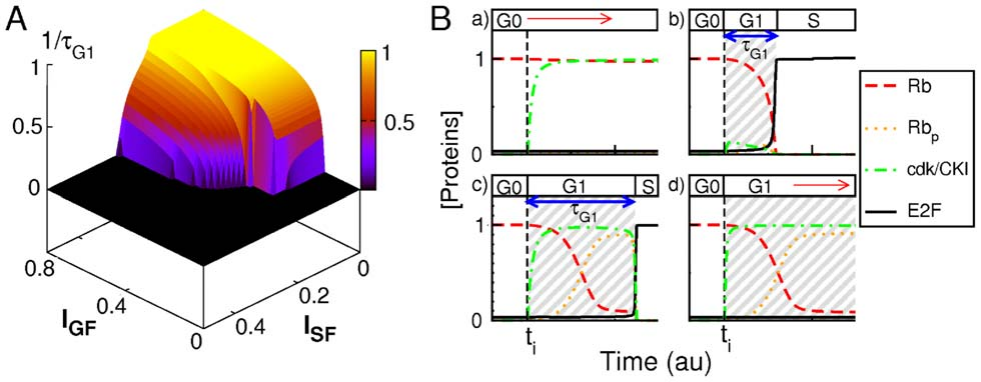
Rate-limiting factors for G1/S transit. (A) Plot depicting the evolution of the rate of G1-phase progression (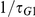) as a function of 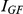 and 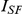 for a simultaneous step of 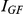 and 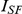 signals. (B) Time-dependent changes in normalized concentration of the main G1 regulatory components following cell exposure to a simultaneous step of 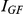 and 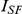 signals at time 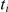. Before that time, 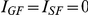 and the cell stands in a G0-like state. Entry into S phase is assessed by the sharp rise of E2F at time 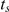. G1-phase duration is defined as the time gap 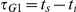. Four combinations of signal intensities are considered: (a) 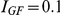, 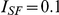; (b) 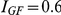, 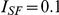; (c) 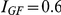, 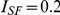; (d) 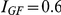, 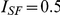. According to the terminology in Table 1, the concentrations shown are: 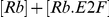 (dashed line), 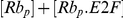 (dotted line), 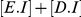 (dash-dotted line), 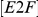 (full line).

For the sake of simplification, it is convenient to consider that G1-phase progression relies on the contrasting activity of only two families of signals: (i) activatory signals, which promote cell division initially by facilitating the accumulation of cyclin D-Cdk4,6 and (ii) inhibitory signals, which oppose cell division by facilitating the accumulation of Cip/Kip proteins. In this case, indeed, the rate of G1-phase is expected to critically depend on the relative levels of the two competing signals. Figure 2 recapitulates how the combination of cell-cycle activatory and inhibitory signals may not only determine the outcome of G1-phase progression but also the timing of G1-phase events. We performed numerical calculations to simulate how G1 phase proceeds in response to a simultaneous step of growth factors and of stress factors applied at time 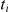. S-phase entry is assumed to occur at time 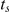, when the concentration of the activator E2Fs becomes larger than half its maximum value. Consistently with previous modeling studies and bifurcation analysis (Fig. S1A), G1/S transition is triggered via a bistable switching process. In this case, bistability is primarily generated by a positive feedback loop through which cyclin E-Cdk2 free the E2F factors from Rb proteins and, thereby, boost cyclin E synthesis and their own accumulation, though other positive feedback loops could possibly also participate in this process [21], [26]. Therefore, G1-phase duration can be defined as the time gap 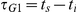.

**Table 1:** Model equations and parameters.

**Differential equations**
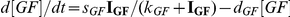
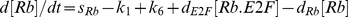
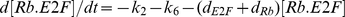
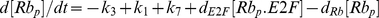
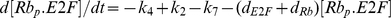
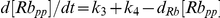
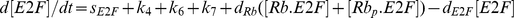
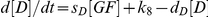
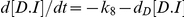
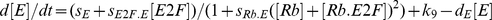
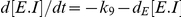
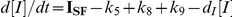
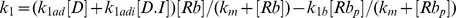
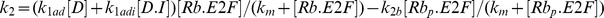
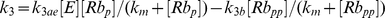
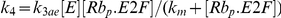
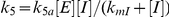
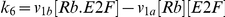
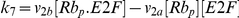
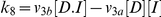
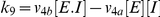
**Dimerization/dissociation kinetic parameters**
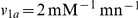 ; 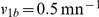 ; 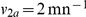 ; 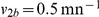 ; 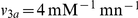 ;
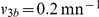 ; 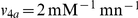 ; 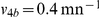
**Phosphorylation/dephosphorylation kinetic parameters**
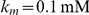 ; 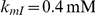 ; 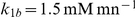 ; 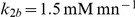 ;
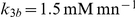 ; 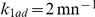 ; 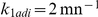 ; 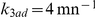 ; 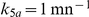
**Synthesis/degradation kinetic parameters**
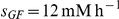 ; 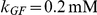 ; 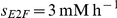 ; 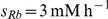 ; 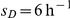 ;
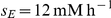 ; 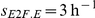 ; 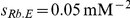 ; 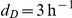 ; 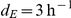 ;
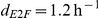 ; 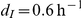 ; 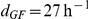 ; 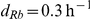

Dynamic equations and parameters associated with the network shown in Figure 1B. There are 12 variables, 31 kinetic parameters and 2 input-dependent *control* parameters (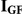 and 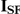). 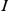: Cip/Kip; 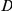: cyclin D-Cdks; 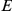: cyclin E-Cdks.

Figure 2A depicts the way how the rate of G1-phase progression (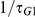) varies as a function of 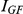 and 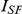. Expectedly, irreversible S-phase entry requires high enough 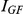 level and low enough 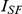 level. Yet, the rate of G1-phase progression does not evolve in the same way as a function of 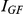 and of 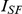 : 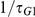 gradually falls when 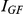 decreases, whereas it first remains nearly constant and, then, slowly decreases when 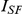 increases. As illustrated in Figure 2B, four main scenarios of G1-phase progression can thus be outlined depending on the relative strengths of the 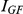 and 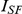 signals. First, below a given 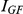 threshold, the network activity remains in a G0-like state, in which Rb proteins are unphosphorylated and the activities of both cyclin E-Cdk2 and E2Fs are low (Fig. 2Ba). Above this threshold, the outcome of G1-phase progression critically depends on the 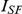 level. At low enough 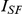 signal, G1-phase progression quickly drifts toward a S-phase entry state associated with a sharp rise in the activities of cyclin E-Cdk2 and E2Fs. Within this scenario, sequential Rb phosphorylation does not occur because the Rb proteins, which are rapidly hyperphosphorylated by cyclin E-Cdk2, fail to accumulate in their hypophosphorylated form (Fig. 2Bb). At higher 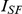 signal, the Cip/Kip proteins accumulate up to a level that becomes sufficient to inhibit the cyclin E-Cdk2 activity without compromising exit from G0, thus enabling sequential Rb phosphorylation to effectively occur and, hence, the accumulation of hypophosphorylated Rb proteins that is required to delay G1 phase progression (Fig. 2Bc). Further 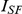 increase above a critical 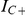 value prevents Rb hyperphosphorylation, thereby blocking S-phase entry (Fig. 2Bd) and setting a stable G1-arrest state in which Rb proteins are steadily hypophosphorylated and the activities of both cyclin E-Cdk2 and E2Fs are kept at relatively low levels.

These simulations bring to the fore that, within the G1 regulatory scheme depicted in Figure 1, Cip/Kip protein stockpiling is instrumental to favor accumulation of hypophosphorylated Rb proteins over that of hyperphosphorylated Rb proteins and, thus, endow mammalian cells with the ability to easily adjust their G1-phase length.

### Identification of regulatory features that contribute to tunable G1 length and reversible G1 arrest

**Figure 3:**
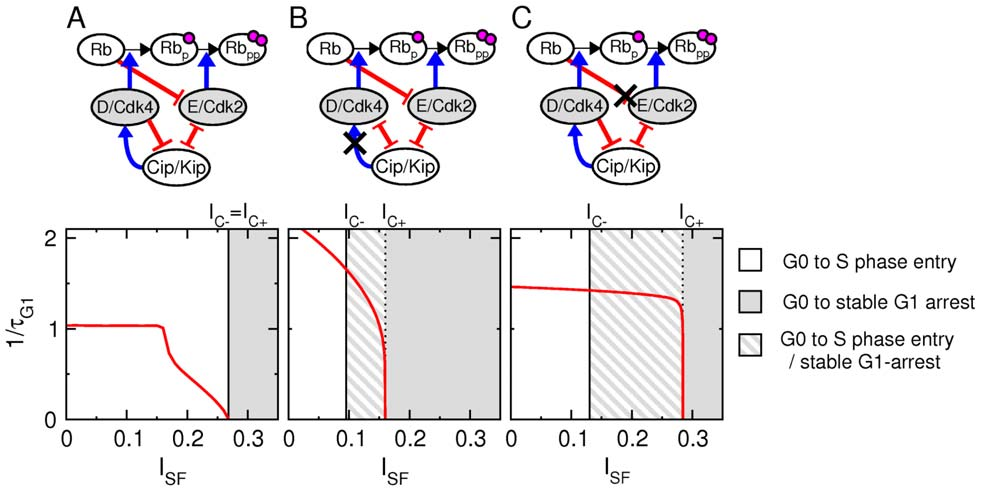
Features of G1-phase regulation responsible for tunable G1 length and reversible G1 arrest. Three different situations have been analysed (Top panels): (A) The standard one corresponding to Figure 1 (see Table 1) and two hypothetical ones in which: (B) the Cip/Kip proteins inhibit the activity of cyclin D-Cdk4,6 (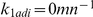, 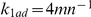) and (C) unphosphorylated Rb proteins does not repress cyclin E transcription (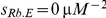 that is compensated by reducing 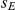 and 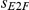 by 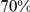). Bottom panels: plots depicting the changes in the rate of G1-phase progression (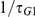) as a function of 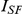, starting from the G0 state (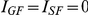), when G0 exit is triggered by an 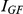 step equal to one (like in Fig. 2). Grey (filled and hatched) regions define 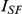 intensities for which the G1-arrest state is stable. Hatched regions bounded by 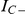 and 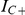 specify 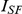 intensities for which G0-arrested cells are able to progress toward S-phase entry following growth factor stimulation but for which G1-arrested cells fail to return to the cell cycle following stress signal withdrawal.

In the previous section, we have shown that stress signal-dependent acccumulation of Cip/Kip proteins can delay S-phase entry and eventually induce a stable G1-arrest state when the 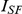 intensity reaches a critical level, 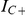. In order to assess whether this cell-cycle arrest is reversible, we performed numerical calculations to simulate how the G1-arrest state evolves when the 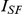 signal is gradually removed. We found that G1-phase progression is restored as soon as 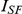 falls below a critical value 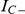 equal to 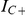, hinting that the G1-arrest state is fully reversible (Fig. 3A).

It was reasonable to hypothesize that the reversible nature of mammalian G1 arrest takes root in the underlying mechanisms of G1-phase regulation, notably in the intricate relationship between the two G1-specific activatory modules and CKIs of the Cip/Kip family. Remind that G1-phase progression is governed by the sequential activation of cyclin D-Cdk4,6 and cyclin E-Cdk2 and that CKIs of the Cip/Kip family exert an opposite effect on cyclin D-Cdk4,6 and cyclin E-Cdk2. In order to test this hypothesis, we performed numerical calculations to simulate how G1-phase duration depends on 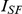 and evaluated 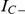 and 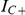 in two distinct hypothetical situations, in which: (1) Cip/Kip proteins inhibit the kinase activity of cyclin D-Cdks (Fig. 3B); (2) cyclin E transcription is not selectively repressed by unphosphorylated Rb (Fig. 3C). In the first situation, the plot depicting the rate of G1-phase progression (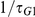) as a function of 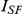 shows that 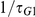 sharply decreases when 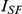 becomes close to 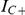. Moreover, G1-phase progression is restored upon stress removal when 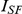 falls below a critical value 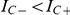, indicating that the G1-arrest state cannot be reversed for 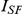 values comprised between 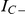 and 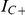. In the second situation, 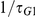 drops even faster when 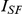 gets close to 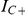 and the 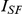 window in which irreversible G1 arrest occurs is still broader (Fig. 3C).

Besides the identification of strategic G1-phase regulatory features, the result of Fig. 3 underscores the existence of two poles apart decision-making scenarios according to whether 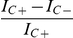 is null (i.e., reversible case) or positive (i.e., irreversible case). To trace back the origin and the significance of these qualitative differences, we also perform bifurcation and sensitivity analysis for the standard and modified G1-phase models (see section A of Text S1). One the one hand, standard bifurcation analysis cannot discriminate between the reversible and irreversible scenarios since bifurcation diagrams are qualitatively similar (compare Fig. S1A and B). On the other hand, sensitivity analysis allows to check that the singular relationship between G1-phase design and decision-making properties does not depend on the precise value of model parameters. Figure S2 shows that 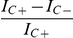 remains either null for the standard G1-phase model or strictly positive for the modified models despite variations of model parameters of about 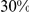 that leads to significant variations of 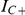 and 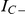 threshold values.

### Dynamical analysis of distinct mechanisms of G1-phase decision

**Figure 4:**
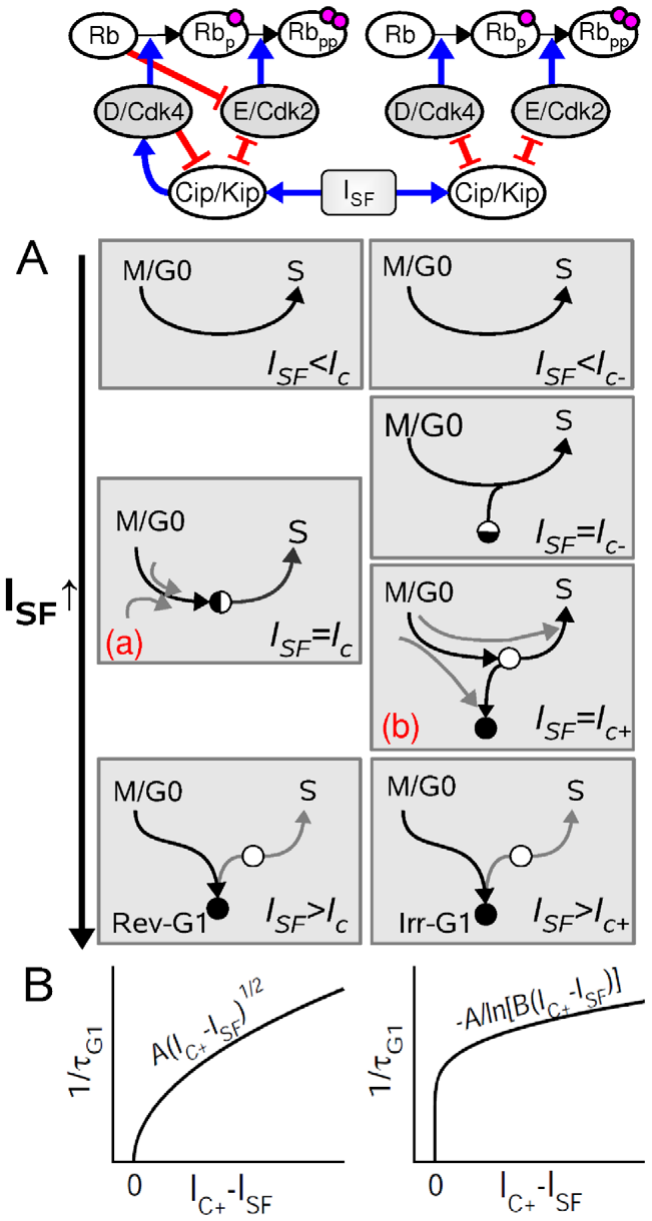
Dynamical mechanisms underlying distinct G1-phase decisions. (A) Schematic representation of how the 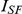 signal modifies the trajectories of G1-phase progression in the state space in the case of reversible (left panels) and irreversible (right panels) G1-arrest states. Black circles and white circles indicate a stable equilibrium linked to a G1-arrest state and an unstable equilibrium, respectively. Half black and half white circle indicates a saddle-node equilibrium. Left and right panels correspond to two qualitatively distinct scenarios. In case of limit cycle trajectories (connecting S to M), panels (a) and (b) would correspond to a saddle-node bifurcation on invariant cycle and a saddle homoclinic bifurcation, respectively. (B) Typical asymptotic relationship between the rate of G1-phase progression (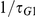) and 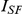 strength associated with reversible and irreversible G1-arrest scenarios (see supporting material).

The dynamical origin of the qualitative differences in G1 length tunability and G1-arrest reversibility unveiled in Figure 3 becomes apparent if one plots schematically the trajectory of G1-phase progression on an appropriate projection of the protein concentration space (Fig. 4). In normal proliferation conditions, the concentrations of the cell-cycle regulatory proteins evolve with time along a limit-cycle trajectory in the high-dimensional protein concentration space, more specifically on the G1-phase portion of a such closed orbit trajectory in our study. Accumulation of Cip/Kip proteins during G1 phase modifies the attractor landscape until, at a critical level of 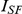 signal, a stable G1-arrest state emerges, typically through a saddle-node bifurcation. Depending on the G1-phase regulatory scheme, however, a stable G1-arrest state can emerge either along or apart from the trajectory of G1-phase progression. Accordingly, the critical level of 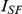 signal, 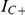, at which the cell-cycle trajectory arising from a mitotic or G0-arrest state converges to a G1-arrest state, may be equal to or larger than the critical level of 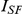 signal, 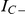, at which the G1-arrest state is stabilized or destabilized. Extrapolating to the full cell cycle, these two examples of cell-cycle exit would correspond to two distinct bifurcation scenarios of limit cycles [27]: (i) a saddle-node bifurcation would occur on an invariant circle (called SNIC bifurcation) when 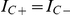; (ii) a saddle homoclinic bifurcation would occur at 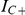 when 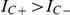.

In these two distinct decision-making scenarios, the relationship between G1 length and 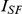 can be captured analytically when the G1 length diverges for 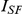 approaching 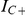 (see section B of Text S1):
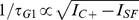
(1)

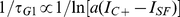
(2)according to whether the dynamical trajectory associated with G1-phase progression passes near a saddle-node ghost (Eq. 1) or near a saddle equilibrium (Eq. 2). These asymptotic laws for G1 length tunability are indeed observed for the standard model and the modified ones when fitting the curves of Figure 3 on a log-log plot (Fig. S3).

### Noisy decision times versus noisy decision fates

**Figure 5:**
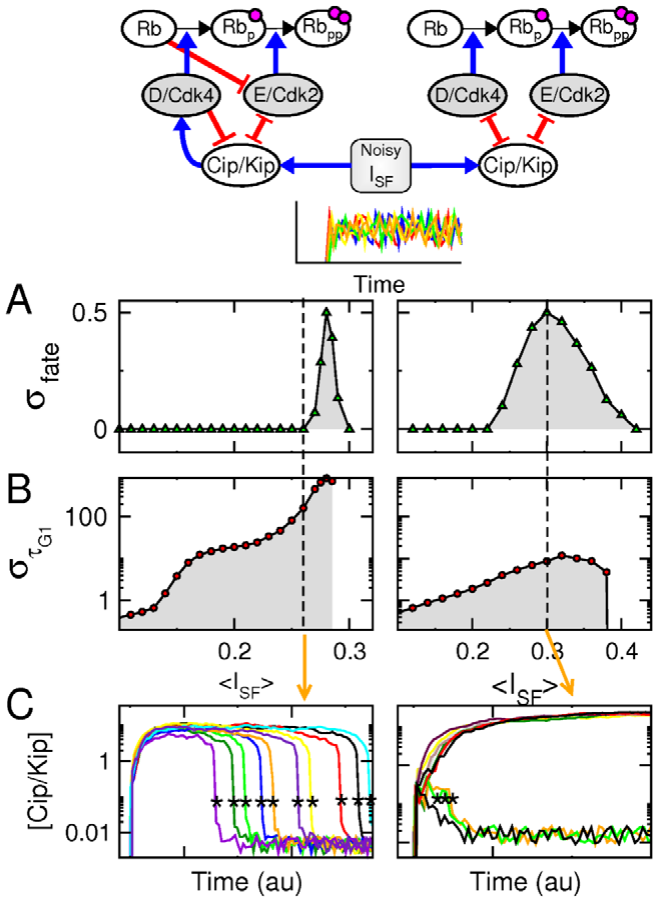
G1-phase decision variability in presence of fluctuating stress signals. (left panels): standard scenario that gives rise to a reversible G1-arrest state and corresponding to the scheme depicted in Figures 1 and 3A. (right panels): scenarios giving rise to irreversible G1 arrest, combining the schemes depicted in Figures 3B and 3C. Numerical simulations were performed on several hundreds of cells subjected to different 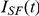 signals with the same mean 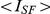 and the same coefficient of variation of 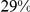. In fact, 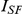 switches every 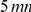 between uniformly distributed random values. (A,B) Plots of 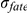 and 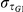, respectively, as a function of 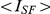. (C) Time course of 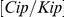 in 10 cells subjected to an average stress input 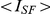 indicated by the dashed line in panels A and B. Asterisks indicate the S-phase entry event (G1/S transition).

Our study thus unveils how subtle differences in the organisation of the G1 regulatory network may nevertheless change drastically the property of G1-phase progression, especially whether G1-arrest state is reversible or irreversible like during senescent or terminally-differentiated state. We show in Figure 5 that cell populations subjected to noisy 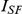 signals statistically behave quite differently depending on the G1-regulatory scheme and G1-arrest strategy that prevail in individual cells. We simulated G1-phase progression in cells subjected to different temporal patterns of 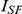 signal characterized nevertheless by the same mean and variance. We then measured the standard deviation 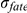 of the decision fates and the standard deviations 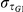 of the decision times, which are defined as followed:
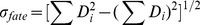
(3)

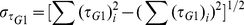
(4)where the 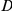 value is equal to 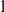, if the final outcome is to enter S-phase, and 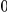, otherwise. For cells displaying the regulation scheme depicted in Figure 3A, all cells are likely to experience the same fate (except in a very small window), either S-phase entry or G1-phase arrest depending on the mean value of 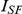 (left panel of Fig. 5A). Yet, when they progress towards S-phase entry, they do so at a highly variable rate (left panels of Fig. 5B and C). Contrastingly, in the situation corresponding to the regulation scheme combining those depicted in Figure 3B and C, cells are prone to experience different fates for a broad range of 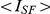 (right panel of Fig. 5A) and those which progress towards S-phase entry tends to display a G1 phase of equal length as assessed by the low 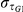 value and the temporal profile of Cip/Kip proteins in various cells (right panels of Figs. 5B and C). It is worth to mention that similar results are obtained by considering intrinsic molecular noises instead of noises in input signals. Thus, fluctuations, extrinsic or intrinsic, can produce either noisy decision times or rather noisy decision fates depending on the particular G1-phase regulatory scheme. Our result suggests that the G1-phase organization of mammalian cells (left panels of Fig. 5) would favor fate reliability over fast decisions, which makes sense since cells in multicellular organisms are exempt from the imperative to divide rapidly and must avoid inappropriate decisions that may perturb the homeostasis of their host as in tumorigenesis.

## Discussion

### Design features of mammalian G1-phase flexibility

Whereas the core cell division process is strongly conserved amongst eukaryotes, entry in and progression through G1 phase follow a highly changeable course depending on cell type and environmental cues. In that phase, mammalian cells are submerged with an abundance of conflicting signals, competing with each other to encourage cell fates as irreconcilable as cell division, differentiation, senescence and death [9], [13]. In that phase also, like in G2, cells are required to repair DNA damages and replication errors committed in S phase. It is not astonishing therefore that mammalian cells have evolved an exceedingly complex web of molecular interactions to control in a contextual manner the length of their G1 phase and the occurrence and timing of its utmost critical issue, which is cell division.

It is reasonable to postulate that the acquired ability of mammalian cells to elaborate flexible G1 phases, especially during developmental processes, takes root in the architecturing of their G1 regulatory network. We therefore developed a dynamical modeling approach with the aim to check the role that could play on G1-phase control two especially striking G1 regulatory elements: (i) the selective transcriptional repression of cyclin E by unphosphorylated Rb proteins in very early G1 phase [14], [15], [36], which operates to delay the apparition of the cyclin E-Cdk2 complexes after growth-factor stimulation; (ii) the opposite effect on cyclin D-Cdk4,6 and cyclin E-Cdk2 of the Cip/Kip proteins, which, following their accumulation in response to stress signals [16]–[18], facilitate the activity of the former complexes whereas they inhibit the activity of the latter ones. Our simulations clearly showed that each one of these two G1 regulatory elements functions to build a sharp frontier between the early and late G1-phase events and restrict the strong mutual antagonism between the Cip/Kip proteins and cyclin E-Cdk2 to a short time window near the G1/S transition. Together, they thus cooperate to endow mammalian cells with the capacity to finely control their rate of progression through G1 phase or to sustain fully reversible G1 arrests. Importantly also, our study proposes that the disruption of these regulatory features through the recruitment of additional regulatory module can easily convert reversible decisions into irreversible ones, which may endow mammalian cells with the ability to control not only the proper timing of their G1-phase decisions but also whether these would be reversible or irreversible.

### Cip/Kip-mediated, exquisitely-sensitive control of G1-phase duration in mammalian cells

Our finding that a moderate increment in Cip/Kip proteins lengthens G1 phase is a priori not surprising and it has already been documented in several experimental studies [39], [40]. Yet, it has not been realized before that the ability of mammalian cells to finely tune their G1 length in response to various constant levels of stress signals and stockpiling of Cip/Kip proteins depends on the contrasting way how distinct G1-specific cyclin-Cdks are regulated by the same entities (unphosphorylated Rb proteins and Cip/Kip proteins), which provides mammalian cells with an extremely powerful avenue to control their rate of G1-phase progression according to both the specificity of cell-cycle inhibitory stimuli and the relative strength of activatory and inhibitory cell-cycle regulatory signals. This result is not only supported by numerical experiments but also by dynamical system analysis that predicts an approximate square-root relationship between the rate of G1 progression and the strength of antimitogenic signals. Although no quantitative data are available to assess this prediction, this could nevertheless account for the huge G1-length variability observed in the course of development in multicellular organisms. In early embryos, cells can proceed through continuous S-M cycles in a mere half hour, paced by the oscillations in the activity of the universal mitosis-specific cyclin-Cdk1 module. As embryogenesis unfolds, however, a G1 delay is incorporated between M and S phases, giving time to cells to integrate a wealth of environnemental signals whose distribution may be spatially organized and, accordingly, to commit to divide at appropriate times in coordination with their neighbours. As a matter of fact, it has been reported that, in embryonic neural and hematopoietic stem cells, the decision whether to differentiate or not, correlates with G1-phase duration [11], [12]. Therefore, the ability of mammalian cells to elaborate exceedingly flexible G1 phases of great variability in length is crucial to generate tissue diversity and ensure coordinated tissue development during embryogenesis [11], [41]. Our study further suggests that this evolutionary capacity may originally stem from the emergence, upon the pressure of environmental constraints, of an early G1-specific cell-cycle activatory module, namely the cyclin D-Cdk module, distinct from the universal mitosis-specific cyclin-Cdk module inherited from unicelled organisms and differently regulated by CKIs.

### Cip/Kip-dependent reversible versus irreversible G1 arrest

It has long been recognized that accumulation of the Cip/Kip proteins in response to genotoxic and cytotoxic stress signals eventually leads to G1 arrest by inhibition of the cyclin E-Cdk2 complexes [42]. Actually, many Cip/Kip-inducing signals have been reported to give rise to reversible G1 arrests, on account of the fact that, upon stress removal, cell cycle progression could be restored [43]–[49]. The rigorous demonstration, however, that such stimuli are truly able to induce fully reversible G1 arrests would require to check whether indeed hysteresis does not occur in experiments in which the stress signal level would gradually be reduced, which is difficult to achieve in practice. Cip/Kip proteins have been acknowledged also to contribute to the establishment of irreversible cell-cycle arrests, for instance in response to differentiation signals or in senescent cells [50]. Our study predicts that, converting Cip/Kip-mediated G1 arrest from reversible to irreversible requires additional modules, besides those included in Figure 1B, to participate in G1-phase regulation. It is noteworthy that human fibroblasts undergoing replicative senescence in culture typically accumulate a number of markers which appear to be causally involved in the onset of senescence, including the p53 tumor suppressor protein and one of its main downstream effector, p21Cip1, but also p16Ink4a [51], [52]. The Ink4a proteins selectively bind Cdk4,6, blocking the assembly of cyclin D-Cdks and, thus, preventing accumulation of cyclin D-Cdks and sequestration of the Cip/Kip proteins in the early stages of G1 phase. According to our study, stress signals favoring the synthesis and accumulation of p16Ink4a, and p21Cip1-inducing stress signals could therefore cooperate to induce irreversible G1 arrests. Interestingly too, it has been reported recently that p27Kip1 fails to inhibit cyclin D-Cdk4,6 only following tyrosine-phosphorylation in its N terminal domain [37], [38]. Thus, context-dependent tyrosine-dephosphorylation of p27Kip1 could offer to mammalian cells a means to shift from a reversible to an irreversible G1-arrest state.

### From cell-cycle models to decision-making theory

A major challenge for science in the twenty-first century is to develop an integrated understanding of how cells and organisms survive and reproduce [53]. In this huge task, modeling approaches that attempt to extract biological design and dynamic principles will certainly prove of great help. Modeling G1-phase regulation is especially appealing for theoreticians because G1 phase is a critical period of the cell cycle during which individual cells make crucial decisions concerning the organism as a whole. In the search for design principles of G1-phase regulation, the present modeling study identifies a subset of singularities that appear to play a paramount role in the temporal control of G1-phase progression but that were dismissed in previous models. It should be kept in mind, however, that the core set of regulations included in our model is embedded within an exceedingly complex web of signalling and regulatory pathways, which work in concert to coordinate cell growth, cell division, cell differentiation, stress management and survival [9]. An important step forward would be to integrate and reconcile together the multitudinous theoretical works that have already analysed in detail one or another aspect of mammalian G1-phase regulation, e.g. the restriction point [20], [22], [25], [26] or the crosstalk between pathways controlling various cell fates [24], [54], [55]. Models of G1-phase regulation are thus an inexhaustible playgroung to investigate decision-making properties in terms of reversibility, timing or stochasticity, which could be extrapolated to other decision-making systems. In particular, the selection between alternative decision strategies - reversible, irreversible or hybrid - may be relevant not only for other cell-cycle arrest decisions [55], [56], but more generally for any biological processes involving sequential choices, such as during cellular differentiation [57], [58], neuronal spiking [59] or brain cognition [60], [61], thereby manifesting universal principles of biological decision making.

## Methods

### Mathematical model equations

The molecular processes subsumed under the G1 regulatory network defined in the first section of the result section and illustrated in Figure 1B are described by a set of differential equations according to the standard principles of biochemical kinetics (Table 1). Thus, the dynamical properties of the mathematical model are are represented by 12 differential equations describing the time-dependent changes in concentrations of individual components of the network occuring following their modification via a variety of biochemical processes including transcriptional activation/repression, translation, degradation, phosphorylation, dephosphorylation, association, dissociation. The phosphorylation/dephosphorylation reactions are supposed to follow the Michaelis-Menten kinetics [21]. A number of assumptions have been made to restrict the quantity of variables: (i) several proteins (e.g, Cdc25, Myc, p53, Ink4) that are sometimes included in other G1-phase models [21], [25], [26], [55], are omitted in our own model because our interest was more specifically focusing on the interplay between, on the one hand, the cyclin D,E-Cdk activatory modules and, on the other hand, the Rb/E2F and Cip/Kip regulatory modules ; (ii) we did not discriminate between the different members of the Cip/Kip, Rb or E2F protein family, which are generally supposed to play similar, redundant roles though in different contexts; (iii) mRNA-regulatory or translocation processes are also disgarded in our model; (iv) the effect of cell growth is neglected as well because cell growth is presumed to have a limited impact on G1-phase progression in somatic cells from multicelled species. The differential equations used to simulate the G1 regulatory network model were integrated using the second-order Runge-Kutta scheme with fixed-time step 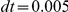.

### Choice of kinetic parameters

Like in most previous models of the cell cycle, the choice of parameters is mostly arbitrary because of the lack of data regarding the rate constants of the physiological reactions that participate in the G1 regulatory network and, also, because we were interested before all on the phenomenological features of the network dynamics. Consistently with the literature, the cyclin half-life is assumed to be shorter than those of the Cip/Kip and E2F proteins that themselves are assumed to be shorter than the half-time of Rb proteins. All parameter values are indicated in Table 1 and their possible changes in the course of the study are specified in the captions. Parameter sensitivity analysis shown in Figure S2 and described in supporting material confirms that the precise choice of kinetic parameters is not critical for the validity and the significance of our results as the qualitative properties of the model are robust to reasonable changes of model parameters.

## Supporting Information

Text S1 **Dynamic analysis of G1-phase models and G1-length tunability.**
(PDF)

Figure S1
**Bifurcation analysis.** Bifurcation diagram showing normalized steady state E2F activity as a function of 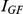 for 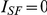 (**A** and **C**) and as a function of 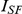 for 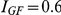 (**B** and **D**). Three G1-phase models depicted in supporting material or in Figure 3 are shown: the model A (**A** and **B**) and the models B and C (respectively blue and red of **C** and **D**). Solid and dashed lines are associated with stable equilibria and saddle equilibria, respectively. White circles highlight saddle-node bifurcation points for which 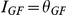 (destabilization of the G0 state) and 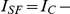 (stabilization of the G1-arrest state).(EPS)

Figure S2
**Parameter sensitivity analysis.** Plot of 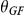, 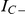 and 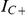 as a function of the normalized hysteresis size 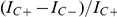 for model A (black crosses), B (red circles) and C (blue squares) in which all model paramaters are multiplied with a factor of 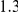 and 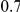.(EPS)

Figure S3
**Asymptotic laws for G1-length tunability.** Plot of 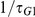 as function of 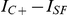 represented in log-log scale for the three G1-phase models: (A) For the model A, the relationship between 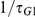 and 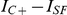 can be fitted with a square root function (with 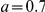) for enough small values of 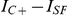. (B) For models B and C, the relationship between 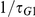 and 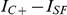 can be fitted with the inverse of a logarithm function (Model B: 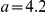 and 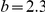; Model C: 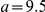 and 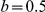).(EPS)

## References

[1] Hartwell LH, Culotti J, Pringle JR, Reid BJ (1974). Genetic control of the cell division cycle in yeast.. Science.

[2] Pardee AB (1974). A restriction point for control of normal animal cell proliferation.. Proc Natl Acad Sci USA.

[3] Blagosklonny M (2003). Cell senescence and hypermitogenic arrest.. EMBO Rep.

[4] Steinman RA, Hoffman B, Iro A, Guillouf C, Liebermann DA (1994). Induction of p21 (WAF-1/CIP1) during differentiation.. Oncogene.

[5] McKinney JD, Cross FR (1995). FAR1 and the G1 phase specificity of cell cycle arrest by mating factor in saccharomyces cerevisiae.. Mol Cell Biol.

[6] Lane ME, Sauer K, Wallace K, Jan YN, Lehner CF (1996). Dacapo, a cyclin-dependent kinase inhibitor, stops cell proliferation during Drosophila development.. Cell.

[7] Fuse T, Yamada K, Asai K, Kato T, Nakanishi M (1996). Heat shock-mediated cell cycle arrest is accompanied by induction of p21 CKI.. Biochem Biophys Res Commun.

[8] Escote X, Zapater M, Clotet J, Posas F (2004). Hog1 mediates cell-cycle arrest in G1 phase by the dual targeting of Sic1.. Nature Cell Biology.

[9] Massagué J (2004). G1 cell-cycle control and cancer.. Nature.

[10] David-Pfeuty T (2006). The flexible evolutionary anchorage-dependent Pardees restriction point of mammalian cells: how its deregulation may lead to cancer.. Biochim Biophys Acta.

[11] Lange C, Calegari F (2010). Cdks and cyclins link G1 length and differentiation of embryonic, neural and hematopoietic stem cells.. Cell Cycle.

[12] Salomoni P, Calegari F (2010). Cell cycle control of mammalian neural stem cells: putting a speed limit on G1.. Trends Cell Biol.

[13] Blomen VA, Boonstra J (2007). Cell fate determination during G1 phase progression.. Cell Mol Life Sci.

[14] Harbour JW, Luo RX, Santi AD, Postigo AA, Dean D (1999). CDK phosphorylation triggers sequential intramolecular interactions that progressively block Rb functions as cells move through G1.. Cell.

[15] Harbour JW, Dean DC (2000). The Rb/E2F pathway: expanding roles and emerging paradigms.. Genes Dev.

[16] LaBaer J, Garrett MD, Stevenson LF, Slingerland JM, Sandhu C (1997). New functional activities for the p21 family of CDK inhibitors.. Genes Dev.

[17] Cheng M, Olivier P, Diehl JA, Fero M, Roussel MF (1999). The p21(Cip1) and p27(Kip1) CDK inhibitors are essential activators of cyclin D-dependent kinases in murine fibroblasts.. EMBO J.

[18] Sherr CJ, Roberts JM (1999). CDK inhibitors: positive and negative regulators of G1-phase pro- gression.. Genes Dev.

[19] Tyson JJ (2002). The dynamics of cell cycle regulation.. Bioessays.

[20] Novak B, Tyson J (2004). A model for restriction point control of the mammalian cell cycle.. J Theor Biol.

[21] Gerard C, Goldbeter A (2010). Temporal organization of cyclin-CDK network of the mammalian cell cycle.. Proc Natl Acad Sci USA.

[22] Aguda BD (1999). The kinetic origins of the restriction point in the mammalian cell cycle.. Cell Prolif.

[23] Swat M, Kel A, Herzel H (2004). Bifurcation analysis of the regulatory modules of the mammalian G1/S transition.. Bioinformatics.

[24] Pfeuty B, David-Pfeuty T, Kaneko K (2008). Underlying principles of cell fate determination during G1 phase of the mammalian cell cycle.. Cell Cycle.

[25] Yao G, Lee TJ, Mori S, Nevins JR, You L (2008). A bistable Rb-E2F switch underlies the restriction point.. Nat Cell Biol.

[26] Yao G, Tan C, West M, Nevins JR, You L (2011). Origin of bistability underlying mammalian cell cycle entry.. Mol Syst Biol.

[27] Guckenheimer J, Holmes P (1983). Nonlinear oscillations, dynamical systems, and bifurcations of vector fields.

[28] Trimarchi JM LJ (2002). Sibling rivalry in the E2F family.. Nat Rev Mol Cell Biol.

[29] Jorgensen P, Tyers M (2004). How cells coordinate growth and division.. Curr Biol.

[30] Csikasz-Nagy A, Battagtokh D, Chen KC, Novak B, Tyson JJ (2006). Analysis of a generic model of eukaryotic cell-cycle regulation.. Biophys J.

[31] Pfeuty B, Kaneko K (2007). Minimal requirements for robust cell size control in eukaryotic cells.. Phys Biol.

[32] Coverley D, Laman H, Laskey RA (2002). Distinct rodna replication complex assembly and activation.. Nat Cell Biol.

[33] Lundberg AS, Weinberg RA (1998). Functional inactivation of the retinoblastoma protein requires sequential modification by at least two distinct cyclin-cdk complexes.. Mol Cell Biol.

[34] Ezhevsky SA, Ho A, Becker-Hapak M, Davis PK, Dowdy SF (2001). Differential regulation of retinoblastoma tumor suppressor protein by G(1) cyclin-dependent kinase complexes in vivo.. Mol Cell Biol.

[35] Cavanaugh AH, Hempel WM, Taylor LJ, Rogalsky V, Todorov G (1995). Activity of RNA polymerase i transcription factor ubf blocked by Rb.. Nature.

[36] Zhang HC, Dean DC (2001). Rb-mediated chromatin structure regulation and transcriptional re- pression.. Oncogene.

[37] James MK, Ray A, Leznova D, Blain SW (2008). Differential modification of p27Kip1 controls its cyclin D-cdk4 inhibitory activity.. Mol Cell Biol.

[38] Blain SW (2008). Switching cyclin D-cdk4 on and off.. Cell Cycle.

[39] Baghdassarian N, Peiretti A, Devaux E, Bryon PA, French M (1999). Involvement of p27Kip1 in the G1- and S/G2-phase lengthening mediated by glucocorticoids.. Blood.

[40] Mitsuhashi T, Aoki Y, Eksioglu YZ, Takahashi T, Bhide PG (2001). Overexpression of p27Kip1 lengthens the G1 phase in a mouse model that targets inducible gene expression to central nervous system progenitor cells.. Proc Natl Acad Sci USA.

[41] Lefresne J, Andéol Y, Signoret J (1998). Evidence for introduction of a variable G1 phase at the midblastula transition during early development in axolotl.. Growth Differ.

[42] Harper JW, Adami GR, Wei N, Keyomarsi K, Elledge SJ (1993). The p21 CDK-interacting protein Cip1 is a potent inhibitor of G1 cyclin-dependent kinases.. Cell.

[43] Hoffman BD, Hanausk-Abel HM, Flint A, Lalande M (1991). A new class of reversible cell cycle inhibitors.. Cytometry.

[44] Ouhibi N, Fulka J, Kanka J, Moor R (1994). A reversible block at the G1/S border during cell cycle progression of mouse embryos.. Int J Dev Biol.

[45] Linke SP, Clarkin KC, Leonardo AD, Tsou A, Wahl GM (1996). A reversible, p53-dependent G0/G1 cell cycle arrest induced by ribonucleotide depletion in the absence of detectable dna damage.. Genes Dev.

[46] Khan SH, Wahl GM (2008). p53 and pRb prevent rereplication in response to microtubule inhibitors by mediating a reversible G1 arrest.. Cancer Res.

[47] Busse D, Doughty RS, Ramsey TT, Russell WE, Price JO (2000). Reversible G(1) arrest induced by inhibition of the epidermal growth factor receptor tyrosine kinase requires up-regulation of p27(KIP1) independent of MAPK activity.. J Biol Chem.

[48] David-Pfeuty T, Nouvian-Dooghe Y, Sirri V, Roussel P, Hernandez-Verdun D (2001). Common and reversible regulation of wild-type p53 function and of ribosomal biogenesis by protein kinases in human cells.. Oncogene.

[49] Pajalunga D, Mazzola A, Salzano AM, Biferi MG, Luca GD (2007). Critical requirement for cell cycle inhibitors in sustaining nonproliferative states.. J Cell Biol.

[50] Zhu J, Woods D, McMahon M, Bishop JM (1998). Senescence of human fibroblasts induced by oncogenic raf.. Genes Dev.

[51] Lundberg AC, Hahn WC, Gupta P, Weinberg RA (2000). Genes involved in senescence and immortalization.. Curr Opin Cell Biol.

[52] Beausejour CM, Krtolica A, Galimi F, Narita M, Lowe SW (2003). Reversal of human cellular senescence: Roles of the p53 and p16 pathways.. EMBO J.

[53] Hartwell LH, Hopfield JJ, Leibler S, Murray AW (1999). From molecular to modular cell biology.. Nature.

[54] Aguda BD, Algar CK (2003). A structural analysis of the qualitative networks regulating the cell cycle and apoptosis.. Cell Cycle.

[55] Toettcher JE, Loewer A, Ostheimer GJ, Yaffe MB, Tidor B (2009). Distinct mechanisms act in concert to mediate cell cycle arrest.. Proc Natl Acad Sci USA.

[56] Pfeuty B, Bodart JF, Blossey R, Lefranc M (2011). A dynamical model of oocyte maturation unveils precisely orchestrated meiotic decisions.. PLoS Comput Biol.

[57] Guantes R, Poyatos JF (2008). Multistable decision switches for flexible control of epigenetic differentiation.. PLoS Comput Biol.

[58] Kuchina A, Espinar L, Garcia-Ojalvo J, Suel GM (2011). Reversible and noisy progression towards a commitment point enables adaptable and reliable cellular decision-making.. PLoS Comput Biol.

[59] Izhikevich M (2006). Dynamical Systems in Neuroscience: The Geometry of Excitability and Burst- ing.

[60] Afraimovich VS, Rabinovich M, Huerta R, Varona P (2008). Transient cognitive dynamics, metasta- bility and decision making.. PLoS Comput Biol.

[61] Gros C (2009). Cognitive computation with autonomously active neural networks: an emerging field.. Cognitive Computation.

