# Supplementary material for: Strategic Cell-Cycle Regulatory Features That Provide Mammalian Cells with Tunable G1 Length and Reversible G1 Arrest
Source: PLoS One. 2012 Apr 23;7(4):e35291. doi: 10.1371/journal.pone.0035291 (PMC3339863; doi:10.1371/journal.pone.0035291)
Supplement: Text S1 — Dynamic analysis of G1-phase models and G1-length tunability. (PDF) [file pone.0035291.s001.pdf]

Supporting Text

Strategic cell-cycle regulatory features that provide mammalian cells with tunable G1 length and reversible G1 arrest

B. Pfeuty

March 19, 2012

1 Dynamic and sensitivity analysis of G1-phase models

A G1-phase model is a model of a specific cell-cycle regulatory module that controls the cell's progression through the Gap1 phase of the cell cycle after the M phase or G0 exit and before the S phase. Although the S phase is typically identified as a transient state in models that describe the entire cell cycle, it is considered as a stable equilibrium in the frame of G1-phase models. In this modular approach, G1-phase progression is thus studied as the input-driven transitory dynamics from a destabilized state toward a stable state, with the possibility that the G1-phase dynamical trajectory slows down or diverts to other attractors upon the receipt of specific inputs. In our study, we restrict our interest to the emergence of a generic G1-arrest state although the specificity of inputs can contribute to discriminate between different types of G1 arrest by recruiting additional regulatory modules.

In order to identify key design principles, properties of G1-phase progression are investigated in three distinct G1-phase models: a standard model (model A) and two modified models that differ from the standard one in a very few regulatory features: (i) the Cip/Kip proteins inhibit the activity of cyclins D-Cdk4,6 instead of upregulating it (Model B); (ii) unphorphosphyrlated Rb does not repress selectively cyclin E transcription (Model C) (see below).

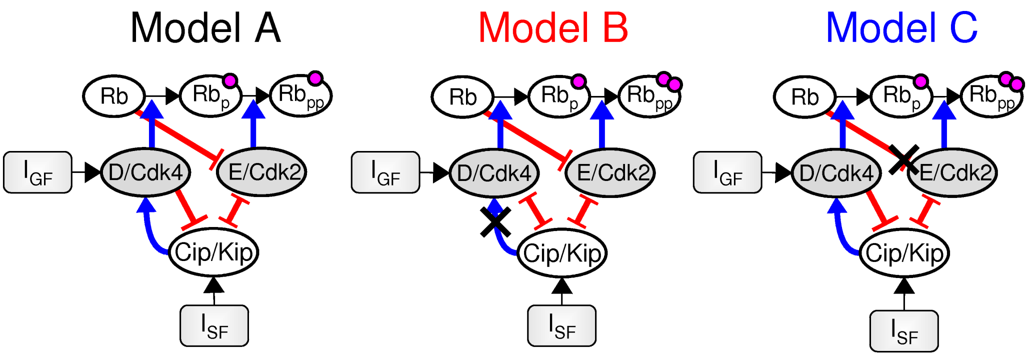

We have shown in the main article (see Fig. 3) that standard G1-phase model and the modified ones display qualitatively different properties of G1-phase progression upon receipt of I_{SF} signals: Model A is characterized by a tunable G1 length and a reversible G1-arrest state whereas models B and C display only short G1 lengths and irreversible G1-arrest states. The present supporting material and figures provide supplemental informations about the origin, the nature and the significance of these differences.

Bifurcation analysis. A preliminary approach to compare the transitory dynamics of these G1-phase models is to plot bifurcation diagrams (Fig. S1), which depict the existence of three steady-state branches associated with the G0, G1-arrest and S-phase entry states, respectively. Saddle-node bifurcation point θ_{GF} (vs I_{C-}) indicates the threshold level of I_{GF} (vs I_{SF}) beyond which the G0 state (vs G1 arrest state) is destabilized (vs stabilized). Bifurcation diagrams of the different G1-phase models display the same bifurcation structure though they slightly differ in the threshold values, θ_{GF} and I_{C-}, at which saddle-node bifurcations occur. Note that the threshold I_{C+} is not apparent in the bifurcation diagrams since these do not show the stable manifold of equilibria. In fact, I_{C+} corresponds to the threshold value of the bifurcation parameter I_{SF} for which the G0 state initial condition is found on the separatrix (stable manifold of saddle equilibrium) between the S-phase entry and G1-arrest attractors.

Parameter sensitivity analysis. To check whether the differences in G1-phase progression observed in the distinct models are relevant and does not depend on the precise values of parameter choosen, we perform parameter sensitivity analysis (Fig. S2). Positive and

negative variations of 30% of all model parameters do not affect the qualitative structure of the bifurcation diagram, but induces only variations of the threshold values I_{C-} and θ_{GF}. More importantly, the property that $I_{C-} = I_{C+}$ in model A and $I_{C-} < I_{C+}$ in model B and C persists despite large parameter variability. We can conclude that not only distinct decision-making strategies are robust to parametric changes, but also the result that distinct G1-phase designs underlie these strategies is relevant irrespective to the precise choice of parameters.

Asymptotic laws for G1-length tunability. Figure 3 of the main article shows clearly that the rate of G1 progression ($1/\tau_{G1}$) vanishes with I_{SF} much more rapidly in models B and C than in model A. Plotting $1/\tau_{G1}$ as a function of $I_{C+} - I_{SF}$ in a log-log representation allows to characterize the asymptotic law for I_{SF} sufficiently close to I_{C+}. For model A, the straight line with a slope of 0.5 indicates that $1/\tau_{G1}$ is proportional to $\sqrt{I_{C+} - I_{SF}}$. For models B and C, the best fit is given for a law of the form: $1/\tau_{G1} = -a/\log[b(I_{C+} - I_{SF})]$. The next section provides a natural explanation based on dynamical system theory for these distinct asymptotic laws for G1-length tunability.

2 Dynamic analysis of G1-length tunability

Let consider the following dynamical system of dimension N that is characterized by a bifurcation diagram similar to those shown in Figure S1:

$$\frac{d\mathbf{x}}{dt} = \mathbf{F}(\mathbf{x}, I_{SF}) \qquad (1)$$

where bold indicates vector and I_{SF} is the main control parameter. In the state space, given an initial point $\mathbf{x_{G0}}$ (i.e., destabilized G0 state), integrating Eq. 1 defines a trajectory γ (i.e., G1-phase progression) that converges to the equilibrium $\mathbf{x_s}$ (i.e., S-phase entry state) in infinite time. We can thus define τ_{G1} (i.e., G1 length) as the time spent on a portion of γ from $\mathbf{x_{G0}}$ to some neighborhood $\tilde{x}_S$ of $\mathbf{x_S}$. When $I_{SF} = I_{C+}$, this trajectory approaches in an infinite time an equilibrium $\mathbf{x_b}$ where the vector field vanishes. For $\delta I = I_{C+} - I_{SF} > 0$, let $\mathbf{x_c}$ be a point of γ where the norm of the velocity vector is minimal. Since $\mathbf{x_c}$ converges to $\mathbf{x_b}$ for $\delta I \to 0$, we can define $\mathcal{B}$, a N-dimensional ball of small and fixed radius, that contains both $\mathbf{x_c}$ and $\mathbf{x_b}$ for δI sufficiently small. τ_{G1} can be thus decomposed into the time intervals, T_I and T_O, respectively spent inside and outside

$\mathcal{B}$. In general, $T_O(\delta I)$ can be expanded as $\tilde{T}_O + 0(\delta I)$. In contrast, T_I diverges when δI tends to zero in a manner that crucially depends on the local structure of the vector field within $\mathcal{B}$. Below is derived analytical expressions of the relationship between τ_{G1} and δI in the two main scenarios (left: saddle-node ghost; right: near saddle equilibrium).

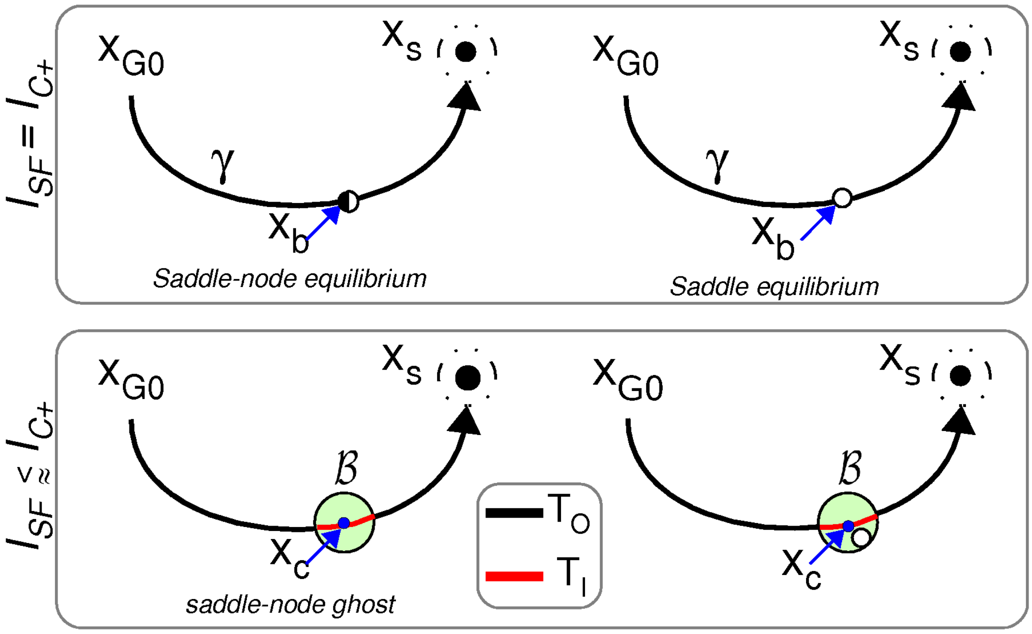

Saddle-node ghost scenario. In this scenario, $\mathbf{x_b}$ is a saddle-node equilibrium. For δI sufficiently small, the dynamics of the multi-dimensional G1-phase model can be reduced within $\mathcal{B}$ to that of the normal form:

$$dx/dt = a_1\delta I + a_2(x - x_{sn})^2 \tag{2}$$

where x is a reduced one-dimensional variable resulting from a non-linear mapping $x = f(\mathbf{x})$. Let thus define x_1 and x_2 as the reduced coordinates for the point of intersection between γ and $\mathcal{B}$. Integrating Eq. 2 between x_1 and x_2 allows to compute how T_I depends on δI:

$$T_I = \frac{\arctan x_2 - \arctan x_1}{\sqrt{a_1 a_2 \delta I}} \tag{3}$$

Given that the time spent by the trajectory γ outside $\mathcal{B}$ remains approximately constant following small changes of δI, τ_{G1} can be approximated at the leading order as:

$$\tau_{G1} \approx \frac{1}{a\sqrt{\delta I}} \tag{4}$$

where a is a parameter that accounts for the characteristics of the non-linear transformation used to derive the normal form.

Near saddle equilibrium scenario. In this scenario, $\mathbf{x_b}$ is a saddle equilibrium. We can use linear theory to compute the trajectory inside $\mathcal{B}$. The saddle equilibrium has one positive eigenvalues (λ_u) and multiple negative ones ($\lambda_{s,j}$). The linearization of the vector field near the saddle point may generically be written in the diagonal form:

$$\frac{dy_j}{dt} = \lambda_s y_j \ , \ j = 1, ..., N-1 \tag{5}$$

$$\frac{dz}{dt} = \lambda_u z \tag{6}$$

where z and y_j axis are tangent to the unstable and a stable manifold of the saddle point. Assuming that the trajectory γ intersects $\mathcal{B}$ with the z-coordinates z_1 and z_2 with $z_1 < z_2$. Integrating Eq. 6 with the boundary condition at $\mathcal{B}$ surface yields:

$$z_1 e^{-\lambda_u} T_I = z_2 \tag{7}$$

Since z_1 typically decreases to 0 proportionally to the bifurcation parameter δI, we can write T_I using Eq. 7 as:

$$T_I = -\frac{1}{\lambda_u} \ln(b_1\, \delta I) \tag{8}$$

where b_1 is a parameter that depends on both local and global features of the vector field. Expressing T_O at the leading order as a constant equal to $\ln(b_2)$, τ_{G1} can be thus approximated as:

$$\tau_{G1} \approx -\frac{1}{\lambda_u} \ln(\tilde{b}\, \delta I) \tag{9}$$

where λ_u is the positive (unstable) eigenvalue of the saddle and the parameter $\tilde{b} = b_1 b_2$ incorporates other disparate properties of the model.
